# Supplementary material for: Optimizing solar drying efficiency: Effects of PCM, and IR on energy and exergy performance
Source: PLoS One. 2025 Jun 17;20(6):e0325042. doi: 10.1371/journal.pone.0325042 (PMC12173389; doi:10.1371/journal.pone.0325042)
Supplement: Attachment — Submitted filename: P-O comments.pdf [file pone.0325042.s001.pdf]

Sincerely,

Dear editor-in-chief, Dr. Morteza Taki

The paper is titled "Optimizing Solar Drying Efficiency: Effects of PCM and IR on Energy and Exergy Performance." I think this study is interesting and original. However, the paper has needed some revisions. The originality of the article is not clearly stated. Differences should be clearly stated with similar studies. If the authors fix the paper, it can be accepted. So, after the article is revised, I need to control it again. Here are some of my comments.

1. The abstract could be expanded to include more detailed information.
2. In the introduction, the authors should connect the state of the art to the goals of their paper by providing a clear and concise analysis. This analysis should identify existing knowledge gaps and relate them to the paper's objectives. Additionally, the authors need to explain the novelty and relevance of their goals. There are numerous relevant papers that should be cited and discussed in this section. However, few studies and articles focusing on the energy-exergy analysis of dryers have been investigated.
3. Enhance the image quality; Figures 2 and 5 can be improved further.
4. The authors should enhance their discussion of the results and compare their findings with those of other relevant research studies.
5. The authors need to include a more comprehensive discussion about the novelty of their paper.
6. Discuss the potential of using this technology in that area in the future.
7. Incorporate recent studies on drying potatoes and compare the findings of this research with those sources. Addressing these suggestions could substantially enhance the manuscript, making it more suitable for publication in the journal.
8. A graphical abstract could be included.
9.

   Here are some tips and questions to consider:
   1) Throughout the text of the manuscript, the term product color quality should replace product quality because the latter includes several factors, and the color change is only one of them.
   2) The author stated that a parabolic collector was used, although it appears to have been implemented as a flat type (Generally, the parabolic design requires a tracking system).
   3) To control the temperature of the dryer, why use a damper rather than a variable-speed fan? A higher mass flow also reduces drying time.
   4) The specific energy consumption was calculated using equation 3, excluding the energy received by the solar collector. This aspect should be revised and recalculated.
   5) Some parameters, such as SEC and EUR, are not mentioned in the text.
   6) Equation 5 does not consider the specific heat of the product or the energy needed to raise its temperature.
   7) In the exergy calculation section, how did you calculate the exergy of the PCM?
   8) Reference No. 15 in line 299 of the examined product is strawberries, not apple slices. Additionally, the mint variety (Mentha spicata L.) used in reference number 16 (line 301) is written incorrectly as Mentha piperita L.

9) The implementation of Phase Change Materials (PCM) is often driven by the need for energy storage and its utilization during periods when solar energy is unavailable. This aspect appears to have been overlooked in this research.
